# Gliosarcoma case report and review of the literature

**DOI:** 10.11604/pamj.2020.35.26.17577

**Published:** 2020-02-03

**Authors:** Awadia Salman Awadalla, Ahmed Mohammed Al Essa, Hassan Hasan Al Ahmadi, Abdulrazaq Al Ojan, Yahya Muazen, Ahmed Alsayyah, Hind Alsaif, Noor Said Alsafwani

**Affiliations:** 1 Department of Pathology, College of Medicine, Imam Abdulrahman Bin Faisal University, Dammam, Saudi Arabia; 2 Department of Neurosurgery, College of Medicine, Imam Abdulrahman Bin Faisal University, Dammam, Saudi Arabia; 3 Department of Radiology, College of Medicine, Imam Abdulrahman Bin Faisal University, Dammam, Saudi Arabia

**Keywords:** Gliosarcoma, parieto-occipital mass, biphasic histology, brain tumor

## Abstract

Gliosarcoma is an unusual subtype of glioblastoma multiforme. Its characteristic features are biphasic configuration, constituting a definite, separate glial and sarcomatous differentiation, on histological evaluation. Herein, we present a rare case of Gliosarcoma that had presented only once in our center in last 13 years. A 60 years old, diabetic, hypertensive male patient came to e emergency department with disturbed level of consciousness and right sided hemiplegia which was progressive over four days. On examination he was, conscious, unoriented in time, person or place, his mouth deviated to left and vitally stable. After initial evaluation, CT scan and MRI were advised. These showed a complex left parieto-occipital heterogeneous mass lesion with cystic and solid components, measuring approximately 5.2x4cm. The mass lesion was seen displacing the occipital horn anteriorly and inferiorly with probable extension into the lateral ventricular cavity. There was no associated midline shift or definite herniation. The lesion was diagnosed as highly suggestive of brain tumor with a differential diagnosis of glioblastoma multiforme or ependymoma. Blood picture revealed a rapidly increasing level of anemia. Surgical intervention comprising left parieto-occipital craniotomy and near total resection of the tumor was carried out. On histopathological and immunohistochemical evaluation the diagnosis of GS was established. A plan of a combination of adjuvant chemotherapy and radiation was formulated that was however, declined by the family. On regular follow up, the patients clinical state rapidly deteriorated with persistence of seizures and requirement of repeated blood transfusions. The patient finally passed away after eighth months.

## Introduction

Gliosarcoma (GS) is an unusual and uncommon type of an aggressive malignancy constituting 2-8% of all primary glioblastomas [1]. It is basically a sub-type of GBM which shows a separate, divergent differentiation into a glial and a sarcomatous component. Stroebe *et al* initially documented this entity in 1895 [2]. It was later described in detail by Feigen *et al* in 1955 [3]. Initially, GS was first considered as a collision of two separate malignant neoplasms, one showing glial and the other a sarcomatous differentiation. The origin of both of these neoplasms was considered to be from vascular cells proliferation. With recent advancement in studies, the current concept of monoclonality has emerged, as genetically both the components have shown to have the same genetic configuration [4-6]. In 2007 edition of classification of tumors by World Health Organization the primary GS has been categorized as a sub type of GBM and it has been attributed a higher grade [7]. It is located mostly in cerebral hemispheres, more so in frontal and temporal lobes. It can also be seen intraventricularly in occasional instances [1,4]. GS has a clinical and radiological overlap with GBM. It shows a predilection for males of around 40-60 years. It imparts a dismal patient’s clinical outcome with an average survival of less than 6 months if patient is untreated [4-9].

## Patient and observation

**Figure 1:**
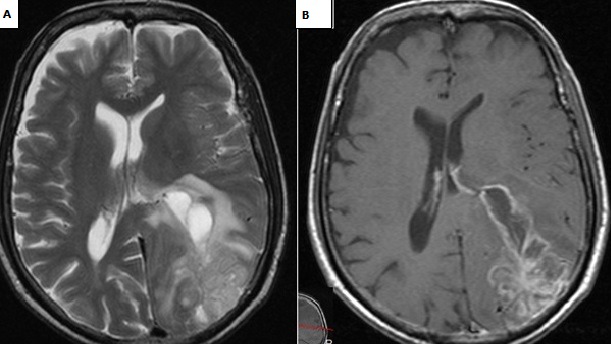
MRI Brain: A) axial; T2W1 B) T1W1 show heterogeneous peripheral left parieto-occipital solid mass with a cystic component; heterogeneous enhancement with rim enhancement seen in B) in keeping with high grade malignancy

**Figure 2:**
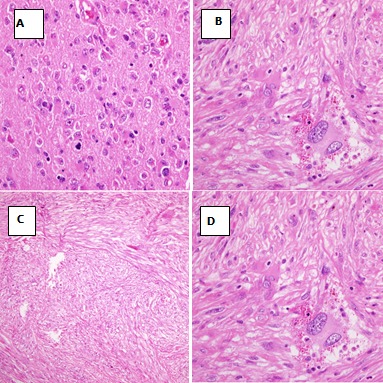
Gliosarcoma: hematoxylin and eosin staining (H&E); A) glial component X20; B) glial component X40; C) sarcomatous component X20; D) sarcomatous component X40

**Figure 3:**
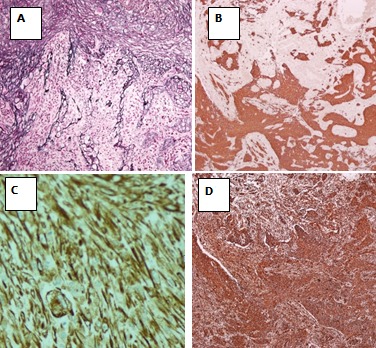
Immunohistochemical and special stains; A) reticulin in sarcomatous component; B) vimentin in sarcomatous component; C) GFAP in glial and sarcomatous component; D) GFAP in glial component

60 years old male, known case of hypertension and diabetes was brought to the emergency department with disturbed level of consciousness and right sided hemiplegia which was progressive over four days. There were no witness’s seizures. On examination, he was afebrile, conscious, not oriented to time, person or place and his mouth deviated to left side but vitally stable. The blood picture was normal besides hemoglobin level which was initially 11g/dl (13-17g/dl) but rapidly came to a low level of 8 g/dl. The power and sensation could not be assessed due to aggressive behavior of the patient. On plain CT scan of the head (Figure 1), there was a complex lesion with cystic and solid components and calcification along left parieto-occipital lobe associated with florid vasogenic edema. A midline shift of 0.6cm was noted without any evidence of hydrocephalus. MRI findings showed left occipitoparietal space occupying lesion with multiple linear projections measuring approximately 5.2x4cm. The mass was heterogeneous with a prominent peripheral rim and vasogenic edema, a cystic component anteriorly; low and bright signal intensity in T1 along with focal hemorrhagic areas in T2. The mass lesion was seen displacing the occipital horn anteriorly and inferiorly with questionable extension into the lateral ventricle. No associated midline sh ift or definite herniation was evident. The impression of MRI was likely glioblastoma multiforme. Presence of probable ventricular extension raised the possibility of an alternative differential diagnosis of ependymoma that was however considered less likely. The surgical intervention consisted of left occipito-parietal craniotomy with almost complete tumor resection. No complications were encountered in the post-operative period. In histopathology, the biopsy was received in neutral-buffered 10% formalin and consisted of multiple fragments of firm, tan-white tissue with lobulated surface, homogenous consistency, measuring in aggregate 4 x 2.5 x 1.5cm. The specimen was totally submitted for histopathological evaluation. Microscopically a biphasic highly cellular neoplasm, comprising a glial component showing anastomosing islands of cells with large round to oval cells with eosinophilic, abundant cytoplasm and large atypical nuclei and prominent nucleoli was seen. This was alternated by a sarcomatous part revealing fascicles of spindle cells with atypical, highly pleomorphic nuclei (some bizarre nuclei) (Figure 2). Immunohistochemical analysis revealed the glial component to be Glial Fibrillary Acidic Protein (GFAP) positive and the sarcomatous component as diffusely positive for reticulin and vimentin but negative for GFAP Figure 3.

## Discussion

Gliosarcoma is an unusual morphological sub type of glioblastoma, with slight predominance in males, age ranging from the sixth to seventh decades and a predilection for the temporal lobes, although it can also affect the frontal, parietal and occipital lobes [1,5,8-10] Several studies concurred that glioblastoma and gliosarcoma are challenging to be distinguished clinically as both have a similar age of presentation, short duration of clinical history and limited post diagnostic life span [1,7,10,11]. There is some controversy regarding the pathogenesis of gliosarcoma. Some authors suggested that the sarcomatous components originated from neoplastic transformation of hyperplastic blood vessels commonly found in high grade gliomas [3,12]. This “collision tumor” concept was supported by early descriptions by Feigin et al of hyperplastic vessels and perivascular arrangement of sarcomatous elements in gliosarcoma [3,5,9]. An alternative theory that has recently gained favor, points to a monoclonal origin of both components of gliosarcoma, with sarcomatous component originating through aberrant mesenchymal differentiation of the malignant glioma [4,11] Reticulin and GFAP helped to distinguish the glial and mesenchymal elements. The sarcomatous areas are reticu¬lin-rich and GFAP-negative, whereas the glial component is reticulin-poor and GFAP-positive [5]. In our case the glial component was GBM with positive GFAP and the sarcomatous was reticulin and vimentin positive. The treatment does not differ from that of glioblastomas, consisting of surgical resection, Depending on patient’s clinical status, radiotherapy and/or chemotherapy may be added [5,6,13]. Patients who receive radiotherapy have a longer life span as compared to patients undergoing surgery exclusively, according to Perry *et al*. [12]. The patient eligible for radiation therapy are younger and present with higher performance status and more favorable prognosis. In our case, a plan of adjuvant therapy of chemo/radiation was disclosed to the family but was refused. On regular follow up the patient clinical condition deteriorated. He developed persistent seizure attacks with increasing requirements for blood transfusions. Unfortunately, the patient expired eighth months later.

## Conclusion

Gliosarcoma, a histological biphasic tumor showing separate glial and sarcomatous components, is a very rare clinicopathological entity that was diagnosed only once in the last 13 years in our center. It is associated with a very aggressive clinical course with patient’s rapid deterioration and poor outcome. The patients mean survival rate averages less than a year even with a combination therapy comprising radio and chemotherapy.
